# The Interactome of Palmitoyl-Protein Thioesterase 1 (PPT1) Affects Neuronal Morphology and Function

**DOI:** 10.3389/fncel.2019.00092

**Published:** 2019-03-13

**Authors:** Tamar Sapir, Michal Segal, Gayane Grigoryan, Karin M. Hansson, Peter James, Menahem Segal, Orly Reiner

**Affiliations:** ^1^Department of Molecular Genetics, Weizmann Institute of Science, Rehovot, Israel; ^2^Department of Neurobiology, Weizmann Institute of Science, Rehovot, Israel; ^3^Department of Immunotechnology, Lund University, Lund, Sweden; ^4^Turku Centre for Biotechnology (BTK), University of Turku, Turku, Finland

**Keywords:** palmitoyl-protein thioesterase 1, PPT1, hippocampal neurons, mass-spectrometry, palmitoylation

## Abstract

Palmitoyl-protein thioesterase 1 (PPT1) is a depalmitoylation enzyme that is mutated in cases of neuronal ceroid lipofuscinosis (NCL). The hallmarks of the disease include progressive neurodegeneration and blindness, as well as seizures. In the current study, we identified 62 high-confident PPT1-binding proteins. These proteins included a self-interaction of PPT1, two V-type ATPases, calcium voltage-gated channels, cytoskeletal proteins and others. Pathway analysis suggested their involvement in seizures and neuronal morphology. We then proceeded to demonstrate that hippocampal neurons from *Ppt1−/−* mice exhibit structural deficits, and further investigated electrophysiology parameters in the hippocampi of mutant mice, both in brain slices and dissociated postnatal primary cultures. Our studies reveal new mechanistic features involved in the pathophysiology of this devastating neurodegenerative disease.

## Introduction

Neuronal ceroid lipofuscinosis (NCL) represents a group of neurodegenerative diseases that usually initiate during early childhood and share accumulation of autofluorescent material in the brain and other tissues (Rider and Rider, 1988; Gupta et al., 2001). The infantile form of the disease (OMIM #256730) is associated with mutations in the *CLN1* gene, that encodes for the palmitoyl protein thioesterase 1 (PPT1) enzyme (Vesa et al., 1995). PPT1 is a thio-esterase, which removes long-chain fatty acids from modified cysteine residues in proteins (Hellsten et al., 1996). The addition of long-chain fatty acids to cysteine residues is also known as *S-*acylation or palmitoylation.

In contrary to other protein modifications involving addition of fatty acids such as myristoylation and isoprenylation, palmitoylation is a reversible and dynamic post-translational modification; the turnover cycle has been estimated to vary between seconds to hours (El-Husseini Ael and Bredt, 2002; Rocks et al., 2010; Shmueli et al., 2010; Ahearn et al., 2011; Fukata et al., 2013; review, Conibear and Davis, 2010). The addition of fatty acids alters the functional properties of proteins at multiple different levels; changes protein-protein and protein lipid interactions, which can affect protein localization as well as protein activity.

Data mining reveal several high confidence subcellular compartments for PPT1 (review, Kollmann et al., 2013). These include membrane rafts, nucleus, lysosomes, the Golgi apparatus, synaptic vesicles, axons and the extracellular region^1^. Our previous work indicated that PPT1 is localized to lysosomes and the endoplasmic reticulum, the nucleus and cilia using transfected cells and endogenous immunostaining (Segal-Salto et al., 2016, 2017). We and others have demonstrated that the protein is secreted using western blot analysis and biochemical assays for PPT1 activity (Camp et al., 1994; Heinonen et al., 2000; Segal-Salto et al., 2016). Protein secretion is indicated also by bioinformatic predictions^2^. In mouse cortical neuron cultures, endogenous PPT1 is targeted to axons in mature neurons and colocalizes with presynaptic markers and axonal varicosities using immunofluorescence and electron microscopy (Lehtovirta et al., 2001; Ahtiainen et al., 2003; Kim et al., 2008). Experiments using transfected neurons largely confirmed these findings (Heinonen et al., 2000; Lehtovirta et al., 2001). Interestingly, Furthermore, over expression of PPT1 has been shown to lead to a reduction in the membrane associated fraction of the palmitoylated GAP-43 (Cho and Dawson, 2000). The palmitoylation of SNAP-25 and GAP-43 was shown to be dependent on an intact secretory pathway, indicated that they are palmitoylated during their transport through the secretory pathway (Gonzalo and Linder, 1998). Post-translational modifications of PPT1, which affect processing and trafficking differ in neuronal and non-neuronal cells (Lyly et al., 2007). Proteomics analyses of synaptosomes have repeatedly demonstrated that PPT1 is part of the synaptosomal proteome (Biesemann et al., 2014; Gonzalez-Lozano et al., 2016; Velasquez et al., 2017). Where one of the data sets included analyses of cortical synaptic membrane proteome of juvenile postnatal days 9 (P9), P15, P21, P27, adolescent (P35) and different adult ages P70, P140 and P280 of C57Bl6/J mice (Gonzalez-Lozano et al., 2016). In that study PPT1 was identified in the synaptosomes in all of the experiments. Collectively, the above data suggests for additional functions for PPT1 outside of lysosomes.

The absence of PPT1 leads to the accumulation of palmitoylated substrates (Lu et al., 1996). Generation of knockout mice for the *Ppt1* gene resulted in a suitable mouse model for the infantile form of neuronal ceroid lipofuscinosis (INCL; Gupta et al., 2001; Jalanko et al., 2005). Experiments conducted in this mouse model indicated that ER stress plays an important role in the neurodegenerative process (Kim et al., 2006a,b; Wei et al., 2008). Nevertheless, it should be noted that some neuronal abnormalities were evident prior to synapse degeneration (Kim et al., 2008). Furthermore, it has been suggested that not only neurons are involved in the pathophysiology but also astrocyte activation is part of the disease (Macauley et al., 2011). We have previously shown that the protein content of membranes extracted from brains of mutant newborn mice (P1) enriched in acylated proteins differed from those derived from wildtype mice. We demonstrated that the majority of the differentially identified proteins were palmitoylated, were associated with neurodegenerative diseases by pathway analysis and were enriched in cilia fractions. We further demonstrated cilia abnormalities in primary cells derived from *Ppt1−/−* mice, mouse embryonic fibroblasts (MEFs), neuroblasts and neurons as well as in brain preparations and suggested that cilia abnormalities should be considered as part of the pathophysiology of CLN1.

In the current study, we identified high-confident PPT1-binding proteins from P0 mouse brain lysates. Pathway analysis indicated that these proteins are involved in neuronal morphology and seizures. Whereas seizures are part of the known pathophysiology of the disease, neuronal structural abnormalities were not documented. We hereby demonstrate that hippocampal neurons from *Ppt1−/−* mice exhibit structural deficits, which led us also to investigate electrophysiology parameters in the hippocampi of mutant mice, both in brain slices and dissociated postnatal primary cultures. Collectively, our studies highlight new mechanistic features involved in the pathophysiology of NCL.

## Materials and Methods

### Mice

Mice mutated for *Ppt1* were previously published (Gupta et al., 2001) and were obtained from Jackson laboratories, each mouse was genotyped using the primers recommended by Jackson^3^. The breeding strategy was heterozygote mating, to avoid a selection bias in the genotype. This study was carried out in accordance with the principles of the Basel Declaration and recommendations of the Animal Welfare Law (Experiment with animals), The Regulation of the Council for experiments with Animals, The Weizmann Institute Regulations (SOP), The Guide for the Care and Use of Lab Animals, National Research Council, 8th edition, The Guidelines for the Care and Use of Mammals in Neuroscience and Behavioral Research, the Institutional Animal Care and Use Committee of the Weizmann Institute of Science. The protocol was approved by the Institutional Animal Care and Use Committee of the Weizmann Institute of Science.

### Antibodies

Anti-GFP (Roche, 11 814 460 001), anti-HA (mouse hybridoma 12CAS), anti-Acetylated tubulin (Clone 6—11B-1, T6793, Sigma), anti-gamma tubulin (Clone GTU-88, Sigma), anti-A cyclase III (C-20, sc-588, Santa Cruz Biotechnology INC., Dallas, TX, USA), anti-Rab3IP (S-14, sn-162069, Santa Cruz Biotechnology Inc., Dallas, TX, USA).

### Cell Culture, Transfections, Immunoprecipitation and Column Generation

HEK293 cells were grown in DMEM medium supplemented with 10% fetal bovine serum (Biological Industries, Kibbutz Beit Haemek, Israel), 100 U/ml penicillin and 0.1 mg/ml streptomycin (Biological Industries, Kibbutz Beit Haemek, Israel) at 37°C 5% CO_2_. Cell were transfected with calcium-phosphosphate as described (ref) with GFP-PPT1 or GFP expression plasmids (previously described, Segal-Salto et al., 2016). Adherent cultured cells were washed in cold PBS and collected by cell scrapers. The cells were collected by low speed centrifugation. Cell pellets were either flash frozen in liquid N_2_ and stored at −80°C for later lysis or were lysed directly by resuspension in lysis buffer containing 150 mM NaCl, 50 mM Tris-HCl (pH 7.4), 5 mM EDTA, 1% Triton X-100 supplemented with Protease Inhibitors (Cocktail, Sigma, Rehovot, Israel). For immunoblots, 10–20 μg of proteins were mixed with SDS sample buffer and separated by SDS-PAGE. The rest of the protein lysates were incubated with appropriate antibodies for 2 h at 4°C. Following this, 15 μl (bed volume) of protein A/G agarose (Santa Cruz, San Diego, CA, USA) pre-blocked in lysis buffer supplemented with 10 mg/ml BSA (Sigma, Rehovot, Israel), was added to each sample for additional 1 h. Immunoprecipitated proteins were pelleted by centrifugation and washed three times in lysis buffer.

**Figure 1:**
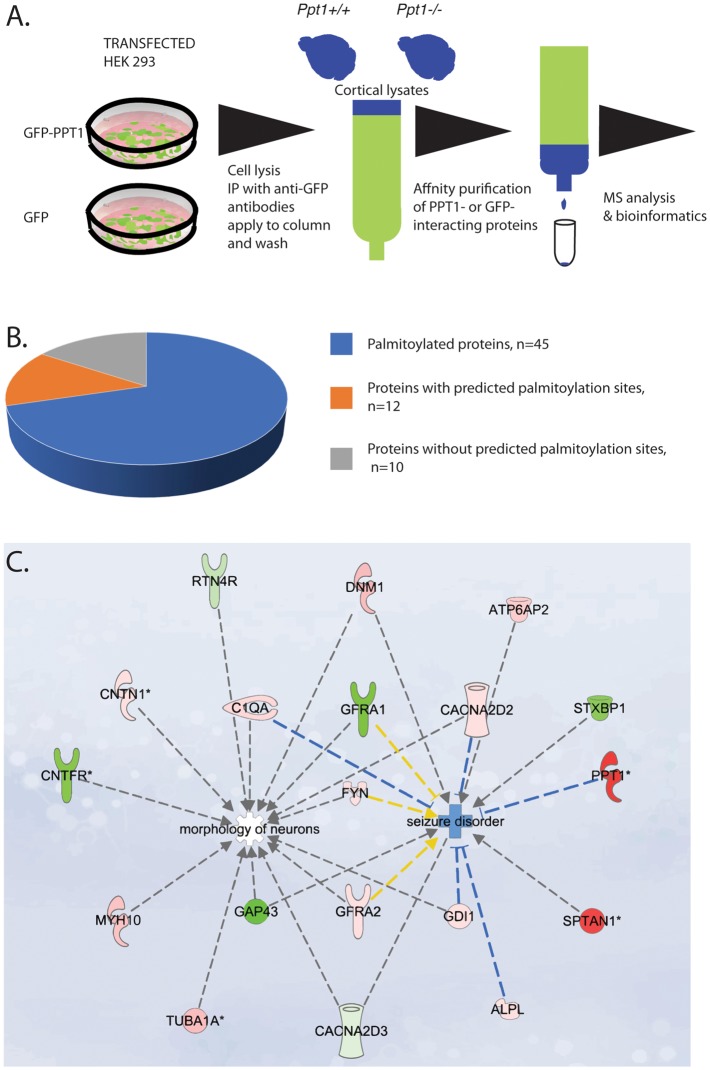
**(A)** Schematic presentation of the proteomics work flow. From left to right: HEK 293 cells were transfected with either GFP-PPT1 or GFP expression plasmids. Cell were lysed, cell lysates were immunoprecipitated with anti-GFP antibodies coupled to protein A/G beads. The beads were loaded onto a column, washed and then protein lysates from P0 cortices derived from either *Ppt1+/+* or *Ppt1−/−* were applied to the GFP- and to the GFP-PPT1- bound beads and then washed. The bound proteins were eluted. The samples were then subjected to MS. The list of the identified proteins was then subjected to bioinformatic analysis. **(B,C)** Characterization of palmitoyl-protein thioesterase 1 (PPT1)-interacting proteins.** (B)** Among the total of 64 PPT1 interacting proteins, all contain at least one cysteine residue, 45 (70%) are known to be palmitoylated, nine (14%) contain sites that are predicted to be palmitoylated. **(C)** Ingenuity pathway analysis revealed that the high confidence proteins may contribute to neuronal morphology and to seizures. Proteins colored in red were enriched in the fractions from *Ppt1−/−* and proteins colored in green were enriched in fractions from wild-type. Note: the origin of PPT1 is from the HEK293 cells used for the initial immunoprecipitation.

PPT1 interacting proteins were identified by differential analysis of proteins detected in a Triton-X-100 soluble fraction (schematic presentation of the process is shown in Figure 1A). The analysis was based on two independent repeats, each repeat included three *Ppt1−/−* and three wild-type brains. Each of the brains was processed individually, half of the lysates from each brain was loaded onto the GFP-PPT1 column and the other half to the GFP column, therefore each brain lysate was subjected both to the experimental column (GFP-PPT1) and the control column (GFP). GFP-PPT1 or GFP proteins were expressed in HEK293 cells. Cells were lysed with lysis buffer [150 mM NaCl, 50 mM Tris-HCl pH 7.4, 5 mM EDTA, 1% Triton X-100 supplemented with Protease Inhibitors (Cocktail, Sigma, Rehovot, Israel)] and immunoprecipitated with anti-GFP antibodies coupled to Protein A/G beads. The washed beads were used to generate a column through which brain lysates from *Ppt1*−/− or wild type were incubated, washed and eluted. Isolation of membranal proteins was done as described previously (Yang et al., 2010). Briefly, brains of neonatal mice (P1) were collected and washed in PBSx1 three times. Brains were homogenized and centrifuged 500× *g* for 5 min to pellet nuclei. The supernatant was centrifuged at 16,000× *g* for 20 min. Following which the membrane pellets were resuspended in buffer A [25 mM 2-(N-Morpholino)-ethanesulfonic acid, 150 mM NaCl, pH 6.5] and then an equal volume of buffer A with 2% Triton-X-100 complemented with phosphatase and protease inhibitors was added. Membranes were incubated for 60 min on ice and were centrifuged at 16,000× *g*, 20 min, 4°C. The lysates were incubated with the GFP or the GFP-PPT1 columns for 1 h and then washed with five volumes of buffer A. The proteins were eluted using protein sample buffer (2.5% SDS, 100 mM DTT, 62.5 mM Tris-HCl pH 6.8, 10% glycerol). The eluted proteins were resolved by 12.5% Criterion precast TGX gels (BioRad, Hercules, CA, USA) and visualized using GelCode Blue Stain reagent (Pierce Biotechnology, Rockford, IL, USA). Each lane was cut into three fractions and each fraction was further divided into smaller pieces (2 × 2 mm), and in-gel digested with trypsin essentially according to the method by Wilm et al., 1996 (Nature 1996, 379, 466–469). Briefly, the gel pieces were de-stained repeatedly in 50% acetonitrile in 50 mM ammonium bicarbonate, and 100 mM ammonium bicarbonate and then dehydrated using 100% acetonitrile and dried in a vacuum centrifuge. The disulfide bonds were reduced with 20 mM DTT in 100 mM ammonium bicarbonate for 1 h at 56°C followed by alkylation with 55 mM iodoacetamide in 100 mM ammonium bicarbonate for 45 min at room temperature in the dark. After washing with 50% acetonitrile in 50 mM ammonium bicarbonate the gel pieces were dehydrated in 100% acetonitrile and dried in a vacuum centrifuge. The gel pieces were rehydrated on ice for 45 min in digestion buffer containing 10 ng/μl sequencing-grade modified trypsin in 50 mM ammonium bicarbonate and then incubated at 37°C overnight. Peptides were subsequently extracted three times with 5% formic acid in 50% acetonitrile for 30 min. Samples were dried down in a vacuum centrifuge and cleaned up using C18 Ultra microspin columns (Nest Group, Southborough, MA, USA) according to the manufacturer’s instructions. The eluted peptides were dried in a vacuum centrifuge and dissolve in 0.1% formic acid. Each of the three fractions was analyzed separately for each sample by nanoflow reversed phase HPLC tandem mass spectrometry.

### Mass Spectrometry

An ESI-LTQ-Orbitrap XL mass spectrometer (Thermo, Bremen, Germany) interfaced with an Eksigent 2D NanoLC system (Eksigent Technologies, Dublin, CA, USA) was used to analyze the peptides derived from in-gel digestions. Peptides were loaded at a constant flow rate of 10 μl/min onto a pre-column (Acclaim PepMap 100, C18, 5 μm, 5 mm × 0.3 mm, Thermo Fischer Scientific, Hägersten, Sweden) and subsequently separated on a 10 μm fused silica emitter, 75 μm × 16 cm (PicoTip Emitter, New Objective, Inc., Woburn, MA, USA) packed in-house with Reprosil-Pur C18-AQ resin (3 μm Dr. Maisch GmbH). Peptides were eluted with a 60 min linear gradient of 3%–35% acetonitrile in water, with a flow rate of 300 nl/min. The LTQ-Orbitrap was operated in a data-dependent mode, simultaneously acquiring MS spectra in the Orbitrap (from m/z 400 to 2,000) and MS/MS spectra in the LTQ. Four MS/MS spectra were acquired using collision-induced dissociation (CID) in the LTQ. The normalized collision energy was set to 35%. Each Orbitrap-MS scan was acquired at 60,000 FWHM nominal resolution settings using the lock mass option (m/z 445.120025) for internal calibration. The dynamic exclusion list was restricted to 500 entries using a repeat count of two with a repeat duration of 20 s and with a maximum retention period of 120 s Precursor ion charge state screening was enabled to select for ions with at least two charges and rejecting ions with undetermined charge state.

### Protein Identification and Quantification

Mass spectrometry raw data files were converted to Mascot Generic Fileformat (MGF^4^) and mzML using Proteowizard version 3.0.3941 (Chambers et al., 2012) and processed further in the Proteios Software Environment (Häkkinen et al., 2009). The MGF files were searched in Mascot (version 2.4.1^4^) against all mouse entries in UniProtKB, downloaded on 2015-08-10, concatenated with reverse sequence decoy entries, totaling 17,0598 protein sequences. Search parameters were: 5 ppm precursor tolerance and 0.5 Da fragment tolerance, one missed cleavage, fixed carbamidomethylation of cysteines and variable oxidation of methionines, default instrument and allowing for one isotope error. The peptide-spectrum-matches (PSMs) were filtered to 0.5% PSM false discovery rate (FDR) in Proteios, corresponding to 2% FDR at the distinct peptide level. Peptide features were detected in the mzML files using Dinosaur (version 1.1.3, Teleman et al., 2016) and matched to MS/MS identifications in Proteios with an m/z tolerance of 0.005 and a retention time tolerance of 0.2 min around feature boundaries. Only MS/MS identifications passing the primary 0.5% PSM FDR cutoff were matched, and subsequently identical identifications in other files were included if they were passing a secondary 10% FDR. Finally, identifications were matched between runs using the Proteios alignment procedure (Sandin et al., 2013) in each fraction (corresponding gel slices), with global alignment recall estimated to >0.99 and global precision estimated to 0.97–0.98 for the three fractions before export of the peptides with integrated intensities as abundance measure.

R (version 3.3.3) was used for subsequent differential expression analysis of peptides in a Jupyter notebook^5^. Abundance values were log2-transformed before pairwise comparisons using LIMMA (version 3.30.13, Ritchie et al., 2015) empirical Bayes between the four biological replicates for each condition. Adjusted *p*-values < 0.1 (10% FDR) were considered significant for subsequent enrichment analysis.

The mass spectrometry proteomics data have been deposited to the ProteomeXchange Consortium *via* the PRIDE (1) partner repository with the dataset identifier PXD006429 and 10.6019/PXD006429.

### Hippocampal Slices

Experiments were conducted according to rules and regulations of Institutional Animal Care and Use Committee of the Weizmann Institute of Science. Transverse slices (400 μm) were collected from the hippocampi of 3–6 month-old male WT and PPT1-KO mice using a McIlwain tissue chopper. Slices were incubated for 1.5 h in carbogenated (5% CO_2_ and 95% O_2_) artificial cerebrospinal fluid (ACSF) at room temperature. The medium contained (in mM) 124 NaCl, 2 KCl, 26 NaHCO_3_, 1.24 KH_2_PO4, 2.5 CaCl_2_, 2 MgSO_4_ and 10 glucose, at pH = 7.4. Recording was made from slices that were slightly submerged in a standard chamber at 33.8–34.0°C with a flow rate of 2.5 ml ACSF/min and full replacement of medium in 6 min, similar to the described in Grigoryan et al. (2012).

Field excitatory postsynaptic potentials (fEPSPs) were recorded in *stratum radiatum* of the CA1 region of hippocampal slices through a glass pipette containing 0.75 M NaCl (4 MΩ). Synaptic responses were evoked by stimulation of the Schaffer collaterals using two sets of bipolar electrodes placed on both sides and equidistant from the recording pipette such that two independent stimulation channels were used for each slice. Tetanic stimulation was delivered at a frequency of 100 Hz using a designated number of pulses. Before applying the tetanic stimulation, evoked fEPSPs (50% of maximum amplitude) were recorded for a stable baseline period of at least 10 min. Tetanic stimulation of one pathway did not cause any noticeable change in response to stimulation of the second pathway, assuring their independance. Data acquisition and off-line analysis were performed using pCLAMP 9.2 (Axon Instruments).

All numerical data are expressed as mean ± SEM, and fEPSP slope changes after tetanic stimulation were calculated with respect to baseline. Statistical comparisons were performed by analyses of variance (ANOVA) tests. *P* values of <0.05 were considered a significant difference between means.

### Primary Hippocampal Cultures

Cultures were prepared as detailed elsewhere (Vlachos et al., 2009; Korkotian et al., 2014). Briefly, pups were decapitated on day of birth (P0–1), their brains removed, the hippocampi were dissected free and placed in a chilled (4°C), oxygenated Leibovitz L15 medium (Gibco) enriched with 0.6% glucose and gentamicin (Sigma, 20 μg/ml). About 10^5^ cells in 1 ml medium were plated in each well, onto a hippocampal glial feeder layer which was grown on the glass for 2 weeks prior to plating of the neurons. Cells were left to grow in the incubator at 37°C, 5% CO_2_ for 2 weeks before use.

### Primary Neurons Imaging and Morphological Measurements

pCAGGS-EGFP or pCAGGS-mCherry plasmids were transferred into hippocampal primary neurons cultured for 6 days (DIV6) on laminin/poly-L-Lysine coated glass by electroporation (EP). Prior to EP, the cells were washed twice with Opti-MEM, and immersed in 300μl DNA solution (10 μg/ μl in Opti-MEM). Cells were subjected to two sets of pulses delivered with a CUY21 Electroporator and CUY900-13-3-5 electrode (NepaGene), were orientated at a different position to maximize treated area coverage. The cultures were kept for an extra week (DIV14) before images were acquired. Automated detection of dendritic trees, and spines, was done using the FilamentTracer tool on Imaris © (Bitplane). Measured values (Spine per segment, Sholl analysis) were extracted from the FilamentTracer tool, and were statistically analyzed on Prism. A segment is defined by the Imaris algorithm as the path between branch points, and between branch points and end points of the traced dendrites. The software recognized branch points and counts the number of spines between adjacent point, i.e., a segment. Imaris (Bitplane) colocalization tool was used to mask SYNCRIP signal outside the spine. The colocalization signal and the total GFP signal were measured using Fiji and the data was subjected to statistical analysis (Prism).

### Immunohistochemistry

Hippocampal cultures at DIV14 were fixed in 4% paraformaldehyde-PBS for 20 min, washed three times with PBS and incubated for 30 min in blocking buffer (10% HS/ FBS, 0.1% Triton X-100 and PBS-Azide (0.05% NaN_3_). Cells were then incubated with primary antibodies (Goat anti PSD-95 Santa Cruz, Rabbit anti SYNCRIP, Ab verified) overnight at 4°C. Cells were washed three times for 5 min each with PBS-T (PBS 0.1% Triton X-100), then incubated with fluorescently-tagged secondary antibodies (Jackson ImmunoResearch) diluted in blocking buffer for 1 h at room temperature in the dark. After three 5 min washes with PBS-T, cells were mounted with ProLong Gold antifade (ThermoFisher Scientific). Images were acquired on a confocal LSM 780 microscope.

#### Electrophysiology

Standard recording medium contained (in mM); NaCl 129, KCl 4, MgCl_2_ 1, CaCl_2_ 2, glucose 10, HEPES 10, pH was adjusted to 7.4 with NaOH, and osmolality to 320 mOsm with sucrose. Tetrodotoxin (TTX, 0.5 μM) and bicuculline (10 μM) were added to the recording medium. Spontaneous synaptic activity was recorded using patch pipettes, as described before (Vlachos et al., 2009). Signals were amplified using a Multiclamp 700B amplifier, accumulated and analyzed with PClamp9. mEPSCs’ amplitude, frequency and rise time were analyzed using Minianalysis software. Statistical comparisons were made with Origin software. Data from 16 to 18 cells were collected from at least four different dissections. *T*-tests were used for comparisons and *P* value of <0.05 was considered significant.

## Results

### PPT1 Interacting Proteins

To identify PPT1 binding proteins, we affinity purified GFP-PPT1 binding proteins using brain lysates derived from *Ppt1−/−* or wild type in comparison with GFP proteins (Figure 1). The source of GFP-PPT1 and GFP proteins was from transfected HEK293 cells expressing the related proteins. From each individual brain Triton X-100 soluble proteins were extracted and half were loaded onto the experimental column (GFP-PPT1) and half were loaded onto the control column (GFP). Our experimental design included two experiments which were conducted at different time points. Each experiment included three *Ppt1−/−* pups and three wild-type pups. Each brain lysate was divided and half was loaded onto the GFP only column and the other half was loaded onto the GFP-PPT1 column. The results of each column load from each brain were analyzed separately. The eluted proteins were subjected to MS analysis followed by bioinformatic analysis. There were between 1004 and 1079 protein groups per sample at an estimated 1% identification FDR, as determined by the target-decoy method in Proteios. Eight thousand seven-hundred and eighty-seven distinct peptides (~2% FDR) were identified after filtering the PSM at 0.5% PSM FDR (a total of 147,550 PSMs passed the threshold). Overall 100 peptides reached statistical significance following comparison with their presence in the control column. The list includes peptides which were statistically significant either in lysates derived from wild type brains and/or in lysates derived from *Ppt1−/−* brains, for 96 of the peptides, protein symbols could be assigned (Supplementary Table S1). There were several proteins for which more than one peptide was identified as significant. Most of the proteins were unique to one set (either wild type or *Ppt1* KO) with the following exceptions: SPTAN1, ATP6V0D1, and BASP1. These three proteins interacted with PPT1 independent of the origin of the protein lysates. Overall, we identified 64 proteins as PPT1 interacting proteins (Supplementary Table S2).

A substantial portion of the identified proteins were previously found to be palmitoylated (Supplementary Table S2, Figure 1B). Two of the identified proteins are well-recognized palmitoyl-proteins, Fyn and Gap43. The other 44 proteins were identified as palmitoylated proteins in one or more of the ~15 palmitoylation-targeted proteomic analyses that have been done to date and additional evidence regarding their palmitoylation will be required. Nevertheless, the current notion is that at present, this group of proteins in under its true representation. Systems analysis of the palmitoyl-proteome screens indicate that at least 10% of the human proteome is susceptible to S-palmitoylation (Blanc et al., 2015), demonstrating that the observed 70% of PPT1 interacting palmitoylated proteins is a significant enrichment. Furthermore, all of the identified proteins contain cysteine residues, which about half of them were computationally predicted to be palmitoylated. Some of the detected proteins are expected to localize extracellularly, including the secreted complement component C1qa, plus other proteins that are expected to tether to the outer surface of the plasma membrane *via* GPI anchors, namely Thy1, Sema7a, Cntn1, Rtn4r, Gpc1, and Alpl. It is possible that PPT1 interacts with these proteins following its secretion.

Next, we subjected the list of the 64 proteins to ingenuity pathway analysis including the observed fold change and the *p* values. Two pathways were identified, one related to seizure disorders and the second related to morphology of neurons (Figure 1C).

### Neuronal Morphology

**Figure 2:**
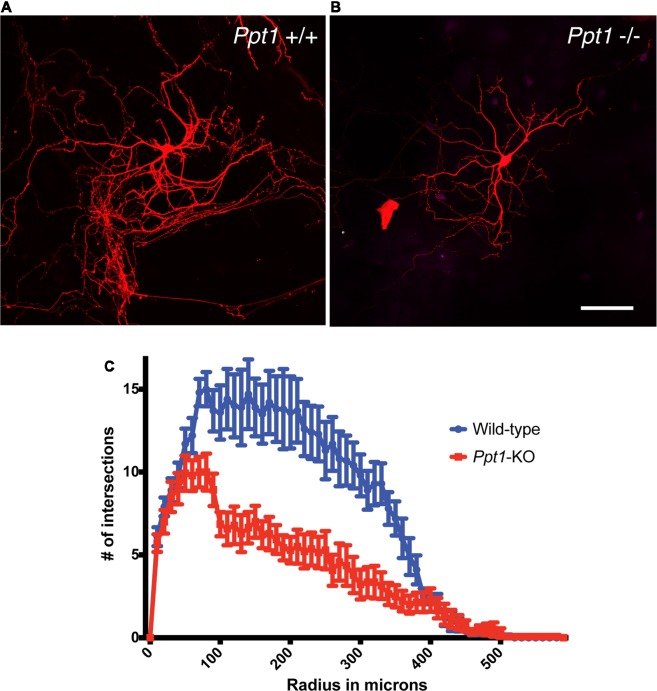
Reduced complexity of cultured *Ppt1*−/− hippocampal neurons. The number of dendrites crossing concentric rings with regular radial increments centered in the neuronal cell body (Sholl analysis) revealed a more simplified dendritic tree in cells lacking Ppt1−/− **(B,C)** in comparison to cells derived from control littermates **(A,C)**. Calibration bar, 100 μM.

The ingenuity pathway analysis revealed a group of high confidence proteins, which are related to cytoskeleton regulation and neuronal morphology, this finding prompt us to investigate neuronal morphology in the absence of PPT1. To identify possible changes in neurons morphology due to the absence of PPT1, we assessed the elaboration of the dendritic tree of primary hippocampal neurons grown in culture. The cells were prepared from littermates, and the cells were transfected with GFP or mCherry expressing plasmids, and visualized after 14 days in culture. The low density of transfected cells allowed semiautomated Sholl analysis, using the FilamentTracer tool in Imaris. Our analysis, revealed a significantly less complex dendritic tree of cells derived from *Ppt1* knockout animals (Figure 2, *n* = 10 cells from each genotype, paired *t*-test *P* < 0.00001, *r* = 0.88).

**Figure 3:**
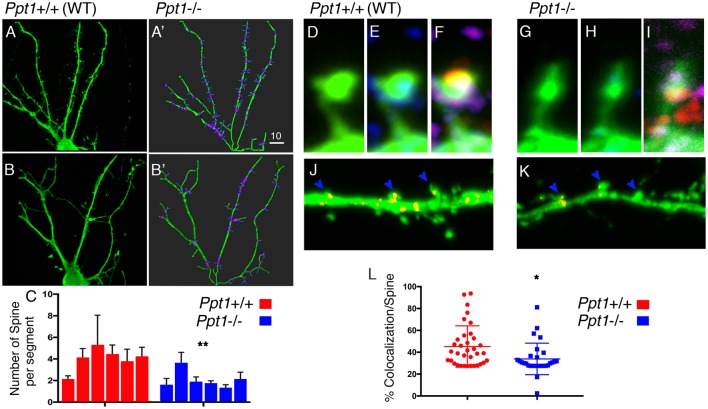
Spine density is modified in *Ppt1−/−* cultured hippocampal neurons. **(A–C)** Hippocampal neurons form control animals **(A,A’)** or *Ppt1−/−* littermates **(B,B’)** were transfected with GFP and grown for 14 days in culture. Dendrites (green) and dendritic spines (blue) were traced using Imaris Filament Tracer tool **(A’,B’)**. **(C)** The numbers of spines branching off dendritic segments were computed from *n* = 6 cells in each group. Averages of the number of spines per each dendritic segment (branch points are highlighted in purple, see “Materials and Methods” section for details) and SEM are plotted, **P = 0.005. **(D–L)** Hippocampal neurons transfected with GFP (green, **D,G**) to mark dendritic spines, and immuno-stained with PSD95 (red, in **E,H**) to mark post-synaptic sites and SYNCRIP (SYP) to locate positive puncta along the dendrites. **(J,K)** SYNCRIP signal (Red) in control or *Ppt1−/−* hippocampal neurons, was masked outside the neurons, showing puncta accumulating in the head of control dendritic spines **(J)** vs. *Ppt1* KO cells **(K)**. **(L)** Colocalization of SYNCRIP in *n* = 36 WT and *n* = 29 *Ppt1* KO dendritic spines, was measured and calculated as percentage of the general GFP signal, *P = 0.0101. The Dot plot chart is depicting all measured spines. Lines indicate average and SEM. Size markers **(A’)** 10 μM, **(G)** 1 μM, **(K)** 2 μM.

The morphological changes detected on a cellular level in cells lacking *Ppt1*, and the possible connection between proteins detected in our initial screen and neuronal activity, prompted us to look in more details at the morphology of the dendritic spines (Figure 3).

Dendritic spines are neuronal potrusions, which receive input, and are essential for synaptic function and plasticity. Spines were visualized using the approach described above but higher resolution images were acquired. Despite a compelling different appearance of the dendritic spine control (Figure 3), only few parameters were found to be significantly different in the two type of neurons. We used the filament statistics tool on Imaris Filament Tracer to automatically compute the number of spines branching off a dendrite in six hippocampal neurons, grown in culture for 14 days, derived from control (Figures 3A,A′) and six neurons from *Ppt1* (Figures 3B,B′) animals. The cells were arbitrarily selected from cultured derived from two control and three *Ppt1* KO mice. We computed the number of spine branch point per dendrite (which is defined as the segment between dendritic branch points and a terminal point), and found that the number of spines branching off a dendritic segment was significantly lower in *Ppt1*−/− primary neurons compared to control cells (3.98 ± 0.43, *n* = 6 vs. 2.03 ± 0.33, *n* = 6, *P* = 0.005). The average length of the dendritic segments did not differ between the control and the *Ppt1*-derived cells (12.1 ± 0.93 vs. 14.4 ± 2.1 μM, *n* = 6, *P* = 0.36 student’s *t*-test). We then examined whether the absence of PPT1 influences the distribution of SYNCRIP/hnRNPQ in the dendritic spines. SYNCRIP is expressed in somatodendritic compartments and is relatively abundant in the hippocampus (Kanai et al., 2004). SYNCRIP is one of the previously identified 58 Triton X-100 soluble proteins that were under-represented in extractions from of *Ppt1* brains (Segal-Salto et al., 2017). In rat hippocampal neurons, SYNCRIP is a component of mRNA granules and is found in punctate expression patterns in the proximal dendrites where it is transported bidirectionally in a microtubule-dependent manner (Bannai et al., 2004). After masking all SYNCRIP signals (Figures 3J,K) outside the dendrite, we were able to measure the percentage of colocalization between the SYNCRIP and GFP out of the general GFP signal, in approximately 30 spines of each genotype. We found that the localization of SYNCRIP puncta within the spines (typically seen at the head of the spines), was significantly lower in cells derived from *Ppt1*−/− animals 33.88 ± 2.675, *n* = 29 vs. 46.84 ± 3.505, *n* = 36, *P* = 0.0046.

### Electrophysiology

**Figure 4:**
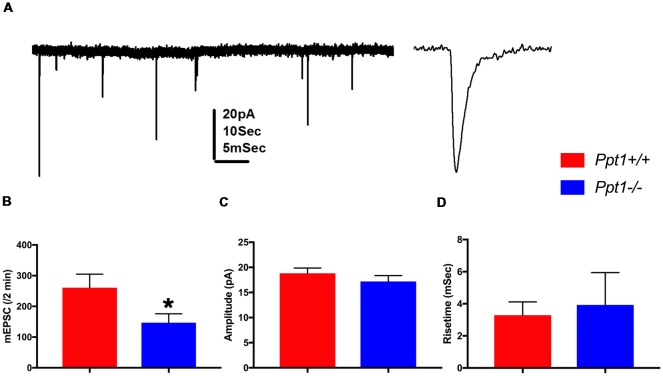
Summary of recordings from wild-type controls and *Ppt1* KO cells. **(A)** Illustration of spontaneous miniature EPSCs recorded from a cultured neuron voltage clamped at −60 mV. Left, a continuous record at low speed, right a zoomed trace taken from the left record. Scale 20 pA for both, 10 s and 5 ms for the left and right traces, respectively. **(B)** Means of discharge rates of 16 controls and 18 *Ppt1−/−* cells. **(A)** Significant reduction in discharge rate is seen (*t*-test, *p < 0.02). **(C,D)** No difference in amplitudes **(C)** or rise time **(D)** between the same two groups is detected.

*Ppt1* KO had a profound effect on the elaboration of the dendritic tree, the spine density and the distribution of SYNCRIP in the spines. We therefore studied in more details the electrophysiological properties of cultured hippocampal neurons from both genotypes. There were no apparent differences in the density of neurons in culture, or passive properties of the recorded neurons between cultured neurons derived from WT or *Ppt1* mutant animals (data not shown). A significant difference was found in the rate of mEPSC, with the *Ppt1* KO cells expressing about 55% of the number of events compared to the wt cells (*n* = 18 and 16 cells, respectively, *p* < 0.02). We could not detect differences in these populations of cells either in the amplitudes of the events, or in their rise time (Figure 4). These findings corroborate the observed reduction in the number of synapses detected in *Ppt1* KO cells mentioned above.

We later searched for alterations in the short- and long-term synaptic plasticity of CA1 fEPSP to afferent stimulation in slices taken from the hippocampi of WT and *Ppt1* KO mice.

**Figure 5:**
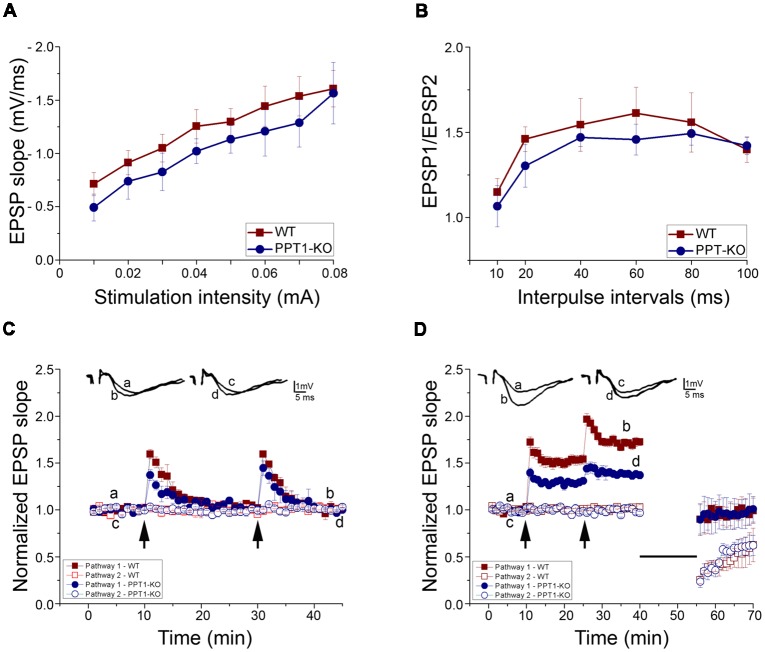
Electrophysiology of transverse hippocampal slices from control and *Ppt1* KO animals. **(A)** Input-output curves of field excitatory postsynaptic potential (fEPSP) slopes for WT and *Ppt1* KO mouse hippocampal slices showing no significant difference between groups. **(B)** Paired-pulse facilitation (PPF) of the fEPSP slopes expressed as response to the 2nd stimulation over the 1st at different interpulse intervals (IPIs; 10, 20, 40, 60, 80, and 100 ms) in WT and PPT1-KO slices. No significant difference in PPF was found. **(C)** Short tetanic stimulation, subthreshold to long-term potentiation (LTP) induction (35 pulses, 100 Hz), was delivered twice to one pathway. The arrows denote the points at which the tetanic stimulation was delivered. The stimulation produced a transient, short term potentiation (STP) which was larger in WT than *Ppt1* KO slices (1.59 ± 0.05 and 1.37 ± 0.06 (*n* = 4 in both groups, *P* < 0.05), correspondingly). **(D)** LTP was induced by high-frequency stimulation, (HFS, 100 Hz, 1 s) at twice the test intensity. The arrows denote the points at which tetanic stimulation was delivered twice to one pathway. The second train produced a further increase in the response. *Ppt1* KO slices expressed a significantly lower magnitude LTP than WT [1.38 ± 0.004 (*n* = 7; *P* < 0.001, *F* value 201.16) compared with 1.71 ± 0.01 (*n* = 7) in WT].

Possible difference in basal synaptic transmission in the WT and *Ppt1* KO slices was assessed from input/output curves. The WT (*n* = 7) and *Ppt1* KO (*n* = 7, slices taken from three different mice each) exhibited similar input/output relations in stratum radiatum of the CA1 region suggesting that basal synaptic transmission is not affected in PPT1-KO mice (Figure 5A).

The form of short-term synaptic plasticity (STP), paired-pulse facilitation (PPF) of fEPSP to afferent stimulation was compared in slices taken from WT and PPT1-KO by using the paired-pulse stimulation (PPS) protocol. Two consecutive stimuli of equal intensity were delivered at varying inter-pulse intervals (IPIs, from 10 to 100 ms) to stratum radiatum. Stimulation of the conditioning pulse was set to evoke a 50% of the maximal EPSP slope. This caused a facilitation of second (test) response in relation to the first response. PPF of fEPSP was not significantly altered in hippocampal slices taken from *Ppt1* KO mice (Figure 5B).

Two short trains of tetanic stimulation, subthreshold to long-term potentiation (LTP) induction (35 pulses, 100 Hz), were delivered to one pathway. This type of stimulation produced only a short lasting post tetanic potentiation (PTP) in both WT and PPT1-KO slices but with a significant difference in their magnitudes (first weak tetanus, 1.37 ± 0.06 times the baseline (*n* = 4; *P* < 0.05, *F* value = 6.15) in PPT1-KO compared with 1.59 ± 0.05 (*n* = 4) in WT; second weak tetanus 1.45 ± 0.08 (*n* = 4) in *Ppt1* KO compared with 1.59 ± 0.02 (*n* = 4; in WT; Figure 5C).

Finally, to study the possible differences in long-term synaptic plasticity in the WT and *Ppt1* KO mice, high- and low frequency stimulation (HFS and LFS, respectively) protocol was used. The LTP generating mechanism was saturated by two successive trains of tetanic stimulation (100 Hz, 1 s, twice of test intensity) applied to one pathway. *Ppt1* KO slices expressed a significantly lower magnitude LTP than WT [1.38 ± 0.004 (*n* = 7; *P* < 0.001, *F* value 201.16) compared with 1.71 ± 0.01 (*n* = 7), respectively, after second tetanus]. LFS (900 pulses, 1 Hz) applied to the previously tetanized pathway led to depotentiation in both WT and *Ppt1* KO slices (0.99 ± 0.01 and 0.97 ± 0.01), respectively. The same pattern of LFS applied to non-tetanized pathway led to decrease of slope of evoked fEPSP (long term depression, LTD) to the level that was similar for both groups (0.61 ± 0.01 in PPT1-KO compared with 0.57 ± 0.02 in WT; Figure 5D).

After the responses stabilized, low-frequency stimulation LFS (900 pulses, 1 Hz, showed on figure as a straight line) was applied to both tetanized and non-tetanized pathways. LFS led to the depotentiation of the previously potentiated pathway and to a LTD of evoked fEPSP of non-tetanized pathway, similarly in both WT and *Ppt1* KO slices.

## Discussion

NCLs, are the most prevalent inherited neurodegenerative diseases (Haltia, 2006). INCL (also known as CLN1), has the earliest age of onset and has the most rapid progression rate. The disease symptoms include progressive visual decline, seizures, motor deficits, cognitive decline, and shortened life span (Nita et al., 2016). As the symptoms progress, the patients suffer from a widespread neurodegeneration and severe retinal and cortical atrophy, the hallmark of NCLs. At least 14 genes have been identified as underlying NCLs and despite different genetic causes, the NCLs disorders share similar clinical and pathological features (Nita et al., 2016). The genetic defect causing INCL are loss of function mutations in the *PPT1* gene, which encodes for PPT1 (Vesa et al., 1995). PPT1 removes palmitic acid from S-acylated proteins *in vitro* but the exact physiological function and the complete repertoire of its substrates is still unknown and the disease mechanism remains elusive. We previously found that the activity of PPT1 is regulated by a positive feedback loop involving palmitoylation/depalmitoylation cycles, and suggested that the palmitate is acting as a noncompetitive inhibitor of PPT1 (Segal-Salto et al., 2016). We later uncovered a novel link between PPT1 deficiency and cilia morphology and function (Segal-Salto et al., 2017). In the current study we continued our effort to shed light on the disease etiology, in particular we set out to record changes that occur in PPT1 deficient neurons. We undertook a non-biased approach aimed at identifying PPT1 binding partners in the mouse brain. By affinity purifying brain lysates on GFP-PPT1 column, while reducing non-specific, GFP binding proteins on a parallel column, we were able to identified 64 PPT1 interacting proteins. Interestingly, all the identified proteins contain at least one cysteine residue, 70% are known to be palmitoylated, and 14% contain sites that are predicted to be palmitoylated. This finding may indicate that some of these proteins, may serve as neuronal substrates for PPT1, however this notion should be verified biochemically. As mentioned above, PPT1 exhibits multiple cellular localizations, it is possible that PPT1 interacts with the observed proteins not only in their known localizations but also upon their delivery to the lysosome following endocytosis or autophagy. We compared our results with several other PPT1-related proteomic data sets; in the data set generated by Scifo et al. (2015) we detected one common protein VCP. In addition, we detected other subunits of SLC25A. In a different data set (Wan et al., 2015), we detected LDHB. An additional study, using PPT1 protein purified from the media of CHO cells identified the F(1)-complex of the ATP synthase (Lyly et al., 2008). In our study, interactions with other ATPases were detected, ATP6AP2, ATP6V0d1, ATP6V1b2. ATP6AP2, has been previously found to be associated with the transmembrane sector of the V-type ATPases (Hein et al., 2015; Huttlin et al., 2015, 2017), and our study also detected two V-type ATPases. Confirming previous results, our data set also detects a self-interaction of PPT1 (Lyly et al., 2007). Subjecting our list of protein to ingenuity pathway analysis pointed at two prominent pathways. First, the software pointed as a link to seizure disorder. Seizures, are a common symptom in the childhood form of NCL (Mole and Cotman, 2015). Infants with the classic infantile CLN1 disease seem to develop normally until 6–12 months. Seizures may occasionally be the first sign of the disease. Over time, affected children suffer mental impairment, worsening seizures, and progressive loss of sight and motor skills. The infantile form of NCL is typically associated with short, sharp muscle contractions called myoclonic seizures, sudden brief jerks of the body and may be limited to the face, extremities, head or involve the entire body.

The second pathway that revealed a functional link between the newly identified PPT1 partners is neuronal morphology. This finding prompt us to look more carefully at possible morphological differences between hippocampal neurons derived from *Ppt1* mutant or wildtype littermates. We found that despite the fact that the neuronal cultures were retrieved from early postnatal pups, prior to the onset of disease symptoms, a remarkable difference in their morphology was noted *in vitro*. We found that the *Ppt1−/−* derived neurons, displayed a simplified structure, with less elaborate and shorter dendritic tree. Of course, we do not exclude the possibility that the accumulation of lysosomal waste contributes to the brain-related phenotypes observed later on. As mentioned earlier, protein palmitoylation represents a common post-translational lipid modification that involves the addition of palmitate, a 16-carbon saturated fatty acid, to specific cysteine residues. Its reversible nature, i.e., palmitoylation/depalmitoylation cycles add a regulatory layer to proteins function. The importance of palmitate cycling for neuronal development and functions have been recognized is several studies (El-Husseini Ael and Bredt, 2002; Huang and El-Husseini, 2005). Among the 64 PPT1 interacting proteins, 44 (including PPT1) were previously identified as synaptosomal proteins. The representation factor was 3.1. A representation factor >1 indicates more overlap than expected of two independent groups. The representation factor is the number of overlapping proteins divided by the expected number of overlapping proteins drawn from two independent groups was 3.1. The probability of this overlap is highly significant, *p* < 2.270e-15.

The first axonal protein that was found to undergo palmitoylation was GAP43, a protein which we recognized in our screen as a PPT1 binding partner. GAP43 is a phosphoprotein associated with the surface of the growth cone. It serves as a substrate for protein kinase C and plays a critical role in axonal growth, regulation of neurite outgrowth and axon pathfinding. The interplay between these two different post-translational modifications is important in determining its localization. Palmitoylation of two cysteine residues at the amino terminus of GAP43 directs it to growth cone membranes. The palmitoylation state of GAP43 is dynamic with high turnover during growth cones outgrowth, and it becomes less palmitoylated as the growth cones nature a stabilize. GAP43 palmitoylation was found to be essential for axon outgrowth (Liu et al., 1993; Gauthier-Kemper et al., 2014). Palmitoylation is often used to target functional proteins to dendritic filopodia and spine membrane compartments. A neuronal specific splice variant of CDC42 undergoes palmitoylation and is sorted to dendritic filopodia and spines (Kang et al., 2008). CDC42 is an actin cytoskeletal regulator that reshapes cellular morphology inducing axon/dendrite outgrowth, dendritic arborization, and spine formation (Wojnacki et al., 2014). We were unable to identify CDC42 as a PPT1 binding protein, however, beta actin (Actb) and actinin alpha 4 (Actn4), that are reported as undergoing palmitoylation (SWISSPALM) were identified in our screen and thus are potential PPT1 substrates. Additionally, αTubulin 1a (Tuba1a) was named as PPT1 binding protein, thus suggesting that cytoskeleton modifications may mediate the morphological effect we see in the absence of PPT1. Spine formation involves transient dendritic filopodia, which can serve as precursors of spines. It is therefore not surprising that a reduction in spine density was associated with less elaborated dendritic tree in PPT1 KO derived neuronal culture. Common molecules that underlie both processes have been identified as undergoing palmitate cycles. Paralemmin, is localized in both dendritic filopodia and spine membranes in developing neurons. Both Paralemmin and CDC42 functionally cooperate during spinogenesis, have the C-terminal palmitoyl motif, and may obey to a common regulatory mechanism (Fukata and Fukata, 2010). Our results indicate abnormal accumulation of SYNCRIP (heterogeneous nuclear ribonuclear protein Q1/NSAP1) in spines of PPT1 KO derived hippocampal neurons. SYNCRIP is an RNA binding protein that targets mRNA to local subcellular sites. SYNCRIP binds specific mRNA including CDC42, N-WASP, and components of the Arp2/3 complex (Arp2/Actr2, Arp3/Actr3, Arpc1a/b, and Arpc4) that encode for regulator of actin polymerization. SYNCRIP was suggested to suppress neurite complexity by modulating local protein expression that effect cytoskeletal dynamics (Chen et al., 2012). The fly homolog of SYNCRIP/HnrnpQ was found to modulate vesicle release in and affect the structure of the synapses in the Drosophila third instar neuromuscular junction (NMJ), suggesting that its mislocalization in the dendritic spines might have more functional implication synaptic transmission (Halstead et al., 2014; McDermott et al., 2014).

Mutations in the human gene DNAJC5/CLN4, that encodes for the presynaptic co-chaperone, CSPα, were shown to cause a late onset NCS. Interestingly, the ANCL pathological mutations in CSPα affect PPT1 expression, localization and enzymatic activity leading to changes in global protein palmitoylation, especially of lysosomal and synaptic proteins (Henderson et al., 2016). CPSα was recognize as one of PPT1 substrates, placing the two proteins in a common disease pathway. DNAJC5/CLN4 patients, accumulate PPT1 in their brains, however the protein is mislocalized and has significantly lower activity. This clinical finding is accompanied by altered palmitome, underscores the importance of PPT1 for neuronal function and survival. Other lines of evidence link PPT1 to neuronal function. In most cell types PPT1 localization is lysosomal, but in neurons it is found also in the presynaptic areas, in axon and in synaptosomes (Lehtovirta et al., 2001; Ahtiainen et al., 2003). The expression of PPT1 in human and mouse brain is developmentally regulated and coincides with cortical synaptogenesis. Most glutamatergic presynaptic boutons contact postsynaptic sites that are located on dendritic spines. Spine morphology is linked to LTP and synaptic strength (Sala and Segal, 2014). Mushroom spines have unique morphological properties that allows compartmentalization. The palmitoylation machinery, and local palmitoylation cycles, are major players in remodeling subsynaptic nano-domains in an activity dependent manner (Fukata et al., 2013). PSD95 is the most abundant postsynaptic scaffolding protein. Its clustering in postsynaptic densities is driven by local palmitoylation of PSD95 by membrane bound palmitoyltransferase, DHHC2 (Fukata et al., 2013) and it is counteracted by serine hydrolases, ABHD17A/B/C (Yokoi et al., 2016). The turn-over of palmitate cycling of PSD95, is rapid but its rate decreases as the synapses develop (Fukata et al., 2013).

Presynaptic and postsynaptic membranes are sites enriched with both palmitoylation/depalmitoylations enzymes as well as their substrates. Glutamate receptor activity regulates palmitoylation of the postsynaptic protein PSD95, and in turn regulates the synaptic retention and removal of AMPARs and thereby the synaptic strength (El-Husseini Ael and Bredt, 2002). This process is thought to be fundamental for synaptic plasticity (Fukata and Fukata, 2010; Fukata et al., 2013).

Taken together, the morphological aspects of PPT1-KO, including the reduced number of dendrites, and fewer and immature dendritic spines, indicate that the cultured neurons of the KO animals are poorer in ability to integrate synaptic information. This is complemented by our electrophysiological results in the cultured neurons, expressing fewer miniature synaptic currents, to indicate that the affected neurons have fewer synaptic contacts with the recorded ones. These results corroborate and extend earlier studies on the role of PPT1 in vesicle recycling and calcium homeostasis (Ahtiainen et al., 2007; Kim et al., 2008). We extended these results to hippocampal slices to find that while the Ppt1-KO slices express somewhat lower input-output relations, they express a dramatic reduction in ability to express LTP in response to tetanic stimulation. Since LTP is considered to underlie an elementary mechanism of learning, our results clearly associate PPT1 with neuronal plasticity, learning and memory, that are severely impaired in the KO animals and humans.

Further experiments are needed to directly relate the deficits in synaptic plasticity with the mechanisms affecting synaptic vesicle recycling, calcium homeostasis and axonal growth and proliferation.

## Data Availability

The mass spectrometry proteomics datasets have been deposited to the ProteomeXchange Consortium *via* the PRIDE (1) partner repository with the dataset identifier PXD006429 and 10.6019/PXD006429.

## Author Contributions

All authors agree to be accountable for the content of the work. TS, MS, GG, KH and MS planned and conducted the experiments. All the above-mentioned authors and PJ, MS and OR were involved in planning of the experiments, data analysis and writing of the manuscript.

## Conflict of Interest Statement

The authors declare that the research was conducted in the absence of any commercial or financial relationships that could be construed as a potential conflict of interest.

## Footnotes

^1^https://compartments.jensenlab.org/Entity?figures=subcell_cell_%%&knowledge=10&textmining=10&predictions=10&type1=9606&type2=-22&id1=ENSP00000394863

^2^https://www.proteinatlas.org/ENSG00000131238-PPT1/tissue

^3^http://www.jax.org/about/index.html

^4^http://www.matrixscience.com

^5^https://jupyter.org
