# Supplementary material for: The Interactome of Palmitoyl-Protein Thioesterase 1 (PPT1) Affects Neuronal Morphology and Function
Source: Front Cell Neurosci. 2019 Mar 13;13:92. doi: 10.3389/fncel.2019.00092 (PMC6424868; doi:10.3389/fncel.2019.00092)
Supplement: Supplementary file 1 [file Table_1.docx]

Symbol External.IDs PPT1.KO-KO_Limma.P.Value PPT1.KO-KO_logFC PPT1.WT-WT_Limma.P.Value PPT1.WT-WT_logFC KO-gfp true PPT1-gfp WT true

| LSAMP | A0A087WP80 | 0.276268723 | 1.404276725 | 0.016044352 | 3.412182788 | 0 | 1 |
| --- | --- | --- | --- | --- | --- | --- | --- |
| LSAMP | A0A087WP80 | 0.655788337 | -0.485554543 | 0.039281478 | 2.530843984 | 0 | 1 |
| GFRA1 | A0A097BVN3 | 0.388539257 | -0.840889093 | 0.030237381 | 2.377434033 | 0 | 1 |
| GAPDH | A0A0A0MQF6 | 0.008218955 | 1.90455776 | 0.315259466 | 0.662384526 | 1 | 0 |
| SPTAN1 | A3KGU5 | 0.686431732 | -0.69111746 | 0.048455423 | 2.79222078 |  | 1 |
| SPTAN1 | A3KGU7 | 0.039693096 | 5.942412392 | 0.900261124 | 0.337180755 | 1 | 0 |
| SPTAN1 | A3KGU7 | 0.968944321 | -0.05191665 | 0.000232922 | 10.73065317 |  | 1 |
| YWHAG | A8IP69 | 0.016086135 | 1.91980232 | 0.335885085 | 0.714473976 | 1 | 0 |
| YWHAG | A8IP69 | 0.010169149 | 2.107316211 | 0.391470698 | 0.644324218 | 1 | 0 |
| TPGS1 | B2RRP1 | 0.043404836 | 1.947370847 | 0.451736147 | 0.636144098 | 1 | 0 |
| ACTB | B2RRX1 | 0.026443383 | 2.429133419 | 0.825548811 | 0.224625645 | 1 | 0 |
| ACTB | B2RRX1 | 0.035344025 | 2.016440655 | 0.850627506 | 0.169255864 | 1 | 0 |
| ALPL | B7XGA6 | 0.536346274 | 0.906351896 | 0.045628361 | 3.194113777 | 0 | 1 |
| TMUB1 | D3Z108 | 0.805302001 | 0.268463947 | 0.042637723 | 2.890661673 | 0 | 1 |
| ECH1 | F7B227 | 0.022487791 | 2.122051066 | 0.342148209 | 0.80129711 | 1 | 0 |
| NBEAL1 | F7D418 | 0.000743143 | 3.998724122 | 0.455828018 | 0.751020889 | 1 | 0 |
| PTPRG | F8VQD7 | 0.810463056 | -0.23502083 | 0.047737486 | 2.173523551 | 0 | 1 |
| GPC2 | F8WHY9 | 0.08087568 | 1.979809065 | 0.011067554 | 2.623109729 | 0 | 1 |
| GPC2 | F8WHY9 | 0.990700441 | -0.011332916 | 0.046515162 | 2.15541864 | 0 | 1 |
| AK9 | G3UYQ4 | 0.000294578 | 2.717172455 | 0.518802375 | 0.399477928 | 1 | 0 |
| STXBP1 | O08599 | 0.185601284 | -0.691546839 | 0.019382443 | 2.144540066 | 0 | 1 |
| GFRA2 | O08842 | 0.526061704 | 0.764355463 | 0.023947342 | 3.379636206 | 0 | 1 |
| GAP43 | P06837 | 0.355797452 | -1.063182029 | 0.028073644 | 2.908359401 | 0 | 1 |
| CNTN1 | P12960 | 0.542624739 | 0.661910545 | 0.014072897 | 2.870023461 | 0 | 1 |
| CNTN1 | P12960 | 0.810723738 | 0.217775793 | 0.047396764 | 2.020711472 | 0 | 1 |
| CNTN1 | P12960 | 0.493649397 | 0.677208365 | 0.044860236 | 2.260831328 | 0 | 1 |
| CNTN1 | P12960 | 0.750658815 | 0.352586203 | 0.044723009 | 2.478142627 | 0 | 1 |
| CNTN1 | P12960 | 0.878673436 | -0.152776287 | 0.01778212 | 3.482733854 |  | 1 |
| LDHB | P16125 | 0.032317367 | 1.625060855 | 0.50666617 | 0.47454712 | 1 | 0 |
| DNM1 | P39053 | 0.033130649 | 2.071883656 | 0.846540548 | -0.183761163 | 1 | 0 |
| FYN | P39688 | 0.859114572 | 0.15218379 | 0.027243205 | 2.185656786 | 0 | 1 |
| GDI1 | P50396 | 0.79261203 | 0.222104588 | 0.003604997 | 3.38769857 | 0 | 1 |
| ATP6V0D1 | P51863 | 0.031122064 | 2.750505458 | 0.175801808 | 1.563265043 | 1 | 0 |
| ATP6V0D1 | P51863 | 0.181495483 | 1.351507219 | 0.031431206 | 2.354556407 | 0 | 1 |
| ATP6V0D1 | P51863 | 0.352025469 | 0.887552789 | 0.039567383 | 2.152762055 | 0 | 1 |
| ATP6V1B2 | P62814 | 0.551897207 | -0.641762166 | 0.026719983 | 2.666849683 | 0 | 1 |
| ATP6V1B2 | P62814 | 0.960120831 | 0.04889133 | 0.030661238 | 2.363597395 | 0 | 1 |
| YWHAZ | P63101 | 0.02478475 | 2.014554904 | 0.723742967 | 0.292433191 | 1 | 0 |
| PHB | P67778 | 0.032743755 | 1.390099638 | 0.281474896 | 0.667094006 | 1 | 0 |
| TUBA1A | P68369 | 0.043848644 | 1.964163329 | 0.371498657 | 0.830364379 | 1 | 0 |
| TUBA1A | P68369 | 0.047635978 | 2.054168917 | 0.422252438 | 0.793472312 | 1 | 0 |
| TUBA1A | P68369 | 0.007504318 | 1.779028851 | 0.878068955 | -0.09061916 | 1 | 0 |
| VCP | Q01853 | 0.593605255 | -0.451238019 | 0.015781902 | 2.924200092 | 0 | 1 |
| MAZ | Q0H9Q2 | 0.036910196 | 1.811446705 | 0.837073769 | 0.196582338 | 1 | 0 |
| ATP6AP2 | Q1XID4 | 0.134268565 | 1.560073287 | 0.045669685 | 2.185254144 | 0 | 1 |
| PPT1 | Q3TEL0 | 8.48E-09 | 5.640026509 | 3.59E-09 | -6.003545856 | 1 | 0 |
| PPT1 | Q3TEL0 | 1.94E-08 | 5.926503161 | 3.03E-09 | -6.709443456 | 1 | 0 |
| PPT1 | Q3TEL0 | 5.19E-10 | 6.313061637 | 5.95E-09 | -5.301104095 | 1 | 0 |
| PPT1 | Q3TEL0 | 1.88E-10 | 6.139072606 | 6.27E-10 | -6.116968959 | 1 | 0 |
| PPT1 | Q3TEL0 | 6.52E-09 | 5.850341408 | 7.86E-08 | -5.461937772 | 1 | 0 |
| PPT1 | Q3TEL0 | 3.18E-09 | 6.482177846 | 6.40E-10 | -7.194997318 | 1 | 0 |
| PPT1 | Q3TEL0 | 8.37E-05 | 3.402058286 | 1.88E-05 | -3.187791533 | 1 | 0 |
| PPT1 | Q3TEL0 | 0.003844866 | 2.610488559 | 0.000281113 | -2.478598658 | 1 | 0 |
| GNB4 | Q3THF3 | 0.020561867 | 1.631764237 | 0.290432701 | 0.752899022 | 1 | 0 |
| PSMA7 | Q3THL2 | 0.03941325 | 1.258237575 | 0.294678545 | 0.611319054 | 1 | 0 |
| GNB1 | Q3TQ70 | 0.014336249 | 1.863572845 | 0.159914365 | 1.007707036 | 1 | 0 |
| GNB1 | Q3TQ70 | 0.026419674 | 1.549594106 | 0.166631001 | 0.923488812 | 1 | 0 |
| PSMA1 | Q3TS44 | 0.035244593 | 2.419485495 | 0.201271277 | 0.968505356 | 1 | 0 |
| PSMD8 | Q3TVY0 | 0.041251813 | 1.413080269 | 0.346278045 | 0.62157278 | 1 | 0 |
| C1QA | Q3TXB1 | 0.018614537 | 1.221372774 | 0.439321923 | 0.373305415 | 1 | 0 |
| OPCML | Q3TYL3 | 0.789101315 | -0.23407244 | 0.040197526 | 2.02866802 | 0 | 1 |
| PAG1 | Q3U1F9 | 0.701350104 | 0.372055625 | 0.034886336 | 2.324695673 | 0 | 1 |
| GPC1 | Q3U379 | 0.234719608 | 1.24743655 | 0.009812004 | 4.24255057 |  | 1 |
| MAPRE1 | Q3U4H0 | 0.046457488 | 1.42009126 | 0.662714956 | 0.294437823 | 1 | 0 |
| RPL10A | Q3U561 | 0.863804267 | 0.100801049 | 0.042746265 | 1.263440529 | 0 | 1 |
| PGAM1 | Q3U7Z6 | 0.003381666 | 3.239582988 | 0.089035821 | 1.480982435 | 1 | 0 |
| PPP1CA | Q3U8W0 | 0.02298503 | 1.635820288 | 0.316427353 | 0.625309447 | 1 | 0 |
| PPP1CA | Q3U8W0 | 0.013090493 | 1.881888134 | 0.5118292 | 0.421172029 | 1 | 0 |
| GNB2 | Q3U9V4 | 0.037922454 | 1.229635887 | 0.102211643 | 0.945017544 | 1 | 0 |
| ACTN4 | Q3ULT2 | 0.047746569 | 1.523395104 | 0.662072906 | 0.377895055 | 1 | 0 |
| CNTFR | Q3UVR2 | 0.483686151 | -0.74513005 | 0.026022703 | 2.705087364 | 0 | 1 |
| CNTFR | Q3UVR2 | 0.950201553 | 0.054396015 | 0.042187191 | 1.998218872 | 0 | 1 |
| THY1 | Q53YX2 | 0.487508395 | 0.678118432 | 0.028721854 | 2.403998689 | 0 | 1 |
| SLC25A5 | Q545A2 | 0.010588896 | 2.157052781 | 0.383510417 | 0.621925536 | 1 | 0 |
| RPS3 | Q5YLW3 | 0.023699328 | 1.458972143 | 0.732841171 | 0.20464436 | 1 | 0 |
| MYH10 | Q61879 | 0.041628084 | 1.863029705 | 0.408813422 | 0.758160653 | 1 | 0 |
| SPTBN1 | Q62261 | 0.562286524 | 0.834580811 | 0.045725594 | 3.178781974 | 0 | 1 |
| KRT73 | Q6NXH9 | 0.340195479 | -0.785648961 | 0.020270033 | 2.094251385 | 0 | 1 |
| CACNA2D2 | Q6PHS9 | 0.674420693 | 0.401293705 | 0.04220305 | 2.16095306 | 0 | 1 |
| IGSF21 | Q7TNR6 | 0.967245571 | 0.049974179 | 0.040892227 | 2.814589688 | 0 | 1 |
| IGSF21 | Q7TNR6 | 0.540068273 | 0.721862975 | 0.016415348 | 3.528196583 | 0 | 1 |
| ERLIN2 | Q8BFZ9 | 0.047515605 | 3.264472445 | 0.544319419 | -0.842006658 | 1 | 0 |
| C12orf56 | Q8CCC3 | 0.002004685 | 1.770208051 | 0.984141391 | 0.009878156 | 1 | 0 |
| IGLON5 | Q8HW98 | 0.479476839 | -0.818531591 | 0.048696412 | 2.521729515 | 0 | 1 |
| IGLON5 | Q8HW98 | 0.475875441 | -0.779500563 | 0.044622169 | 2.436890301 | 0 | 1 |
| OLFM1 | Q8R357 | 0.644419374 | -0.417804316 | 0.033005968 | 2.194408913 | 0 | 1 |
| SFPQ | Q8VIJ6 | 0.046282875 | 1.812985515 | 0.99518322 | 0.004775361 | 1 | 0 |
| BASP1 | Q91XV3 | 0.03298677 | 2.513090508 | 0.016239545 | 2.415829601 | 1 | 1 |
| BASP1 | Q91XV3 | 0.021813232 | 3.048040747 | 0.838021547 | -0.215832461 | 1 | 0 |
| BASP1 | Q91XV3 | 0.212121495 | 1.187956234 | 0.037483147 | 2.117972038 | 0 | 1 |
| RTN4R | Q99PI8 | 0.754439974 | -0.302970997 | 0.025204673 | 2.508746492 | 0 | 1 |
| TMTC2 | Q9CX93 | 0.842296342 | 0.185703264 | 0.004923063 | 3.597378622 | 0 | 1 |
| SEMA7A | Q9QUR8 | 0.453957603 | -0.78969347 | 0.035846654 | 2.56955659 | 0 | 1 |
| SEMA7A | Q9QUR8 | 0.813341764 | -0.250022543 | 0.041803963 | 2.431427639 | 0 | 1 |
| SEMA7A | Q9QUR8 | 0.894572386 | 0.1428758 | 0.031351212 | 2.660621684 | 0 | 1 |
| CACNA2D3 | Q9Z1L5 | 0.996595055 | -0.004303778 | 0.010617988 | 3.906189201 |  | 1 |

Supplementary Table 1: List of peptides annotated to proteins found to be statistically significant in the comparison using either lysates of brains derived from *Ppt1-/+* or wild-type controls.
