# Supplementary material for: The Interactome of Palmitoyl-Protein Thioesterase 1 (PPT1) Affects Neuronal Morphology and Function
Source: Front Cell Neurosci. 2019 Mar 13;13:92. doi: 10.3389/fncel.2019.00092 (PMC6424868; doi:10.3389/fncel.2019.00092)
Supplement: Supplementary file 2 [file Table_2.docx]

| Symbol | Palmitoylated: https://swisspalm.org/proteins | Predicted sites (cysteines) |
| --- | --- | --- |
| Actb | Y |  |
| Actn4 | Y |  |
| Ak9 | N | N(25) |
| Alpl | Y |  |
| Atp6ap2 | Y |  |
| Atp6v0d1 | Y |  |
| Atp6v1b2 | Y |  |
| Basp1 | Y |  |
| C1qa | Y |  |
| Cacna2d2 | Y |  |
| Cacna2d3 | Y |  |
| Cntfr | Y |  |
| Cntn1 | Y |  |
| Dnm1 | Y |  |
| Ech1 | Y |  |
| Erlin2 | Y |  |
| Fyn | Y |  |
| Gap43 | Y |  |
| Gapdh | Y |  |
| Gdi1 | Y |  |
| Gfra1 | N | N(31) |
| Gfra2 | N | Y (32) |
| Gnb1 | Y |  |
| Gnb2 | Y |  |
| Gnb4 | Y |  |
| Gpc1 | N | Y(19) |
| Gpc2 | N | Y(15) |
| Iglon5 | N | N(7) |
| Igsf21 | N | N(6) |
| Krt73 | N | Y(11) |
| Ldhb | Y |  |
| Lsamp | N | Y(7) |
| Mapre1 | Y |  |
| Maz | N | Y(17) |
| Myh10 | Y |  |
| Nbeal1 | N | N(6) |
| Olfm1 | N | N(7) |
| Opcml | Y |  |
| Pag1 | Y |  |
| Pgam1 | Y |  |
| Phb | Y |  |
| Ppp1ca | Y |  |
| Ppt1 | Y |  |
| Psma1 | Y |  |
| Psma7 | Y |  |
| Psmd8 | Y |  |
| Ptprg | Y |  |
| Rpl10a | Y |  |
| Rps3 | N | N(3) |
| Rtn4r | N | Y(16) |
| Sema7a | N | N(18) |
| Sfpq | Y |  |
| Slc25a5 | Y |  |
| Sptan1 | Y |  |
| Sptbn1 | Y |  |
| Stxbp1 | Y |  |
| Thy1 | Y |  |
| Tmtc2 | N | Y(5) |
| Tmub1 | N | N(17) |
| Tpgs1 | N | Y(5) |
| Tuba1a | Y |  |
| Vcp | N | N(12) |
| Ywhag | Y |  |
| Ywhaz | Y |  |
| 64 proteins | 45 Palmitoylated |  |

Supplementary table 2: The list of PPT1 interacting proteins, whether palmitoylated and for unknown the number of cysteine residues.
